# *Brucella *spp. infection in large ruminants in an endemic area of Egypt: cross-sectional study investigating seroprevalence, risk factors and livestock owner's knowledge, attitudes and practices (KAPs)

**DOI:** 10.1186/1471-2458-11-341

**Published:** 2011-05-19

**Authors:** Hannah R Holt, Mahmoud M Eltholth, Yamen M Hegazy, Wael F El-Tras, Ahmed A Tayel, Javier Guitian

**Affiliations:** 1 Veterinary Epidemiology and Public Health Group, Department of Veterinary Clinical Sciences, Royal Veterinary College, London, UK; 2 Department of Hygiene and Preventive Medicine (Zoonoses), Faculty of Veterinary Medicine, Kafrelsheikh University, Kafrelsheikh, Egypt; 3 Department of Animal Medicine, Faculty of Veterinary Medicine, Kafrelsheikh University, Kafrelsheikh, Egypt; 4 Genetic Engineering and Biotechnology Research Institute, Menufiya University, El-Sadat, Egypt

## Abstract

**Background:**

Brucellosis is regarded as one of the major zoonotic infections worldwide. It was first reported in Egypt in 1939 and is now endemic, the predominate species of *Brucella *in cattle and buffalo in Egypt is *B. melitensis*. The aim of the study was to estimate seroprevalence of *Brucella *spp. in cattle and buffalo reared in households in an Egyptian village, identify risk factors for animals testing seropositive and to assess the knowledge, attitudes and practices (KAPs) of livestock owners with regards to brucellosis.

**Methods:**

A cross-sectional study was carried out in a village in Menufiya Governorate of Egypt. In June and July 2009, 107 households were selected using systematic sample and all lactating cattle and buffalo present in the household were sampled and tested for antibodies against *Brucella *spp. In addition, a questionnaire collecting information on potential risk factors for *Brucella *spp. infection in cattle and buffalo was administered to the household member responsible for rearing the livestock. Between December 2009 and February 2010 households were revisited and a second questionnaire regarding KAPs associated with brucellosis was administered.

**Results:**

True individual and household seroprevalence were estimated to be 11.0% (95% CI: 3.06% to 18.4%) and 15.5% (95% CI: 6.61% to 24.7%), respectively. Cattle and buffalo kept in a household with sheep and goats had 6.32 (95% CI: 1.44 to 27.9) times the odds of testing seropositive for *Brucella *spp., compared to cattle and buffalo that were not. Most participants in the study stated that livestock owners assist in the parturition of ruminants without wearing gloves and that some farmers sell animals which they suspect are *Brucella *infected to butchers or at market. Many participants made their livestock's milk into cheese and other dairy products without pasteurising it.

**Conclusions:**

Brucellosis was endemic at high levels, in the current study. Although livestock owners had good general knowledge of brucellosis, they still appeared to participate in high-risk behaviours, which may contribute to the high seroprevalence in the area. Veterinarians, public health authorities and other community leaders need to collaborate to control the disease in animals and to manage the risk of human exposure.

## Background

Egypt has the largest buffalo population in the Middle East and buffalo and cow's milk are the third and sixth most valuable of the food and agricultural products of Egypt, respectively [1]. More than 70% of Egypt's total livestock population is owned by small holders, who keep a few cattle and buffalo in their household as a source of milk and dairy products for home consumption or to sell, often unpasteurized, in local markets [2,3].

The WHO considers brucellosis to be a neglected zoonosis because, despite its widespread distribution and effects on multiple species, it is not prioritised by national and international health systems [4]. It is caused by gram-negative bacteria of the genus *Brucella *which show strong host preference [5]. The species of *Brucella *which infect livestock and their primary hosts are *B. melitensis *(sheep and goats), *B. abortus *(cattle), *B. suis *(pigs) and *B. ovis *(sheep) [6,7]. Brucellosis decreases productivity of infected livestock by causing abortions, reducing fertility and decreasing milk yield, resulting in substantial economic losses [8,9].

Brucellosis was first reported in Egypt in 1939 and is now considered endemic in most parts of the country [10]. Despite its economic and public health importance, in recent years, the official Egyptian brucellosis control programme does not appear to have been fully implemented [10,11]. Hegazy et al (2009) estimated that between 1995 and 2006 less than 7% of all female livestock were tested for brucellosis in Kafrelsheikh Governorate in any given year. Furthermore, little has been done to control brucellosis in small ruminants, which has lead to the transmission of *B. melitensis *into cattle and buffalo populations of Egypt [10].

Although good quality estimates of the prevalence of brucellosis in Egypt are scarce, recent studies in some areas of the Nile Delta region suggest the prevalence in large ruminants is high; in Kafrelsheikh Governorate in 2009 12% (95% CI 8.7% to 15.4%) of cattle milk tanks and 14% (95% CI 10.1% to 17.9%) of buffalo milk tanks were seropositive for *Brucella *spp., although it is unknown how many positive animals contribute to these milk tanks [12]. *B. melitensis *biovar 3 is currently the most common isolate of *Brucella *in Egypt [10,13]. *B. melitensis *poses the biggest public health threat due to its high pathogenicity and infectiousness [14].

In 2007 a population-based survey of acute febrile illness patients was conducted in Fayoum, a rural governorate of Egypt, from the results of the survey human incidence of brucellosis was estimated to be 64 and 70 per 100 000 people in 2002 and 2003, respectively. If incidence estimates in the study had been based purely on hospital admissions, they would have been 3.8 per 100 000 [15]. Brucellosis is considered an occupational hazard; risk factors identified for human brucellosis in the study were close contact with animals, exposure to aborted materials and consumption of dairy products. As close contact with animals is common in rural areas of Egypt and healthcare is not easily accessible for rural populations, rural residents are likely to have a high risk of brucellosis. The most effective way of reducing incidence in humans, is by controlling brucellosis in livestock [16,17].

Recently, farmer's attitudes and husbandry practices have received more attention as important factors influencing the spread of animal disease [18,19]. Assessing knowledge, attitudes and practices for other diseases, such as avian influenza (AI), has been helpful for policy makers to develop control strategies and health education campaigns [20]. To date, there is no information on the knowledge, attitude and practices (KAPs) associated with brucellosis in Egypt, however studies in Kenya and Saudi Arabia have suggested that awareness of the disease and its routes of transmission among livestock keepers may, on occasions, be poor [21,22].

The aim of the current study was to investigate the epidemiology of brucellosis in an Egyptian village. The study objectives were i) to estimate seroprevalence of *Brucella *spp. in cattle and buffaloes at the individual animal, and household level ii) to identify risk factors for cattle and buffalo testing seropositive for *Brucella *spp. and iii) to assess livestock owners KAPs regarding brucellosis. Based on previous work in Egypt and the Middle East, it was hypothesised that keeping cattle and buffaloes in a household with sheep and goats would be a risk factor for brucellosis, as these species are the primary hosts of *B. melitensis*, which is currently the predominant species of *Brucella *present in Egypt.

## Methods

### Study design and study population

This study was conducted in a village in Menufiya Governorate in the Nile Delta region of Egypt, the village was selected due to convenience. The study population comprised all households with lactating cattle and buffalo in the village and the study was carried out in two phases. Firstly, a cross sectional study was carried out during June 2009 to estimate the seroprevalence of *Brucella *spp. in cattle and buffalo at the household and animal level, and to identify risk factors for seropositive status against *Brucella *spp. In phase two of the study, a survey of the KAPs of livestock owners with regards to brucellosis was conducted between December 2009 and February 2010. The study was approved by the Ethics and Welfare committee of the Royal Veterinary College, London, UK and complied with local legislation and the Helsinki Declaration.

### Sampling Strategy

As there was no sampling frame in the village, households were selected for inclusion in the study using systematic sampling, within each of the selected household's herd all lactating cattle and buffalo were sampled. Target sample size for simple random sampling was calculated to be 138 individual animals, for an expected prevalence of 10%, 95% confidence and 5% precision. In order to account for clustering within households this was multiplied by a design effect of 1.2, which was calculated using expert knowledge, assuming an average of two animals would be lactating per household and an intracluster correlation coefficient of 0.2. The target sample size was therefore 166 individual animals, or 83 households. As the number of households in the village was estimated to be 2000 a sampling interval of 24 was used.

One road leading away from the mosque, which is the centre point of the village, was selected at random; along this road one household was randomly selected to be the starting point for the subsequent systematic selection of households. Every 24^th ^household along this road, and all side streets leading from it, was selected. Once the outskirts of the village were reached sampling continued clockwise until another road was reached, this road was then taken back towards the centre of the village, whilst continually sampling households. Next the road opposite the first road sampled was taken, and sampling continued in this manner.

In each selected household occupants were asked if any cattle or buffaloes were kept in the household and if any were currently lactating. If a household did not have any cattle or buffalo or none were lactating at the time of the study the neighbouring house was visited, until a household with lactating cattle and/or buffaloes was found. Once a suitable household was found the occupants were asked for oral consent to take part in a "livestock disease" study, it was not specified that the disease of interest was brucellosis, as this may have biased the results of the study.

### Data collection, sample collection and laboratory technique

On the first visit, a pre-tested structured questionnaire designed to collect information regarding potential risk factors for cattle and buffalo becoming infected with *Brucella *spp. was administered to the member of the household in charge of rearing the livestock. All interviews were carried out by the senior author and a local veterinarian in the evening, when animals are normally brought in from the fields for milking.

During this initial visit, the member of the household who was responsible for milking the animals was asked to collect two milk samples directly into a sterile 50 ml container. Five ml of formalin (10%) was added to one sample immediately after collection and this sample was refrigerated until testing, one to five days after collection, the other sample was frozen. The milk samples were tested for antibodies to *Brucella *spp. using a commercial indirect ELISA (BRUCELISA, Veterinary Laboratories Agency, UK). Testing was carried out in the commercial El-Ahram laboratory in Tanta, Egypt. The manufactures instructions for performing the ELISA where strictly adhered to, the cut-off value for classifying samples as positive/negative was calculated as 10% of the mean optical density of eight positive control wells and was 0.062.

During December 2009 (6 months after the initial visit) a health professional familiar with the village revisited the same households. On this visit a two-part questionnaire designed to collect information on villager's KAPs regarding brucellosis was administered. This questionnaire was developed in English and independently reviewed before being translated into Arabic. The first part of the questionnaire was administered to the household member in charge of rearing the livestock and the second part of the questionnaire was directed at household members responsible for processing dairy products. Participant's knowledge of brucellosis was assessed with regards to awareness of brucellosis, species affected and routes of transmission. Attitudes and practices related to brucellosis were assessed by investigating the management of livestock and processing and consumption of dairy products, in order to identify potential routes of transmission of *Brucella *spp. between livestock and from livestock to humans.

Upon completion of the questionnaire, the interviewer provided household members with relevant disease information and gave the occupants of the households the opportunity to ask questions about brucellosis. Disease information included a description of the disease in animals e.g. symptoms, the potential routes of human exposure and measures to prevent infection in animals and humans. The visitor also advised on the appropriate actions if they suspected an animal was infected and encouraged participants to cooperate in any brucellosis vaccination or testing programmes.

A copy of both questionnaires is available from the corresponding author upon request.

### Seroprevalence estimation using simulation modelling

Simulation modelling was used to estimate true individual and true household seroprevalence of *Brucella *spp. in the village. The models were stochastic in order to account for sample size and inadequate performance of the diagnostic test (sensitivity and specificity < 100%). The variables and parameters included in the models are summarised in Table 1.

**Table 1:** Definition of the variables and parameters used in the models to estimate true seroprevalence of *Brucella *spp. in a village of the Nile Delta, Egypt

Variable	Definition	Distribution, Value or Equation used	Data Source
***Se***	Individual test sensitivity	Triangular: (0.952, 0.98, 0.996)	Manufacturer's estimates were used as the most likely values and minimum and maximum values were obtained from previous studies validating the ELISA test
***Sp***	Individual test specificity	Triangular: (0.881, 0.985, 0.991)	

***S***	Number of animals which tested seropositive for *Brucella *spp.	22	Results of serological tests

***N***	Number of individual animals tested	151	Results of sampling

***AP***	Apparent seroprevalence	Beta(23, 130)	Results of sampling/testing (α = *s *+ 1, β = *n *- s + 1)
***h***	Number of cattle and buffalo sampled within a household	1, 2, 3 or 4	Results of sampling

***I***	Number of infected animals in a household	Discrete(*h *= 2;75,25) (*h *= 3;70,20,10) (*h *= 4;70,15,10,5)	Estimated from the number of test positive animals within households, can range from 1-*h*

***U***	Number of uninfected animals in an infected household	*U = h-*I	Test results, could range from 1 to 3.

***P(+/+)***	Probability that at least one infected animal within a household tests positive	*P(+/+) = 1-(1-Se)^I*	Outputs of previous distributions

***P(-/+)***	Probability that, in an infected household, at least one uninfected animal tests positive	*P(-/+) = 1-(1-Sp)^U*	Outputs of previous distributions

***HSe***	Probability that a positive household is correctly identified i.e. household sensitivity	*HSe = P (+/+) + P(-/+)*	Results of previous Equations

***HSp***	Probability that a negative household is correctly classified i.e. household specificity	*HSp = Sp^h*	Results of previous Equations

***s***_***h***_	Number of households of size *h *where at least one animal tested seropositive	11(*h = *2), 1(*h = *3), 0(*h = *4)	Results of serological tests

***n***_***h***_	Total number of households of size *h *sampled	40(*h = *2), 2(*h = *3), 1(*h = *4)	Results of sampling

***AHP***_***h***_	Apparent household seroprevalence of households of size *h*	Beta (*s*_*h *_+ 1, *n*_*h *_- *s *+ 1)	Results of sampling/tests

The true individual seroprevalence (*TP*) of *Brucella *spp. in cattle and buffalo in the village was estimated from distributions of apparent prevalence (*AP*) and test sensitivity (*Se*) and specificity (*Sp*) [23];

Households were classified as positive when at least one cattle or buffalo tested seropositive for *Brucella *spp. As the number of animals sampled within a household ranged from one to four, true household seroprevalence was initially estimated separately for each strata (household size = one, two, three or four). True household seroprevalence for households with one lactating animal was estimated identically to true individual seroprevalence. For households with two or more lactating animals true household seroprevalence (*THP*) was estimated from distributions of household sensitivity (*HSe*) and specificity (*HSp*) as opposed to individual *Se *and *Sp *of the test. Outputs for *HSe *and *HSp *were combined to estimate *THP *for each stratum [23]:

A mean of the results of true household-level seroprevalence, weighted in accordance with the sample size in each stratum, was calculated to estimate overall true household seroprevalence. Both models (individual animal and household) were run for 10,000 iterations using Monte-Carlo simulation to obtain probability distributions of true individual animal and household seroprevalence in the village.

### Risk Factor Analysis

Quantitative variables were categorised into quartiles or quintiles to account for departures from normality and univariate analysis was performed with individual cattle and buffalo as the unit and serological status for *Brucella *spp. (positive vs. negative) as the outcome. Odds ratios for the association between each explanatory variable and *Brucella *serological status were calculated and chi-square tests were performed to assess the significance of the association. Explanatory variables were assessed for collinearity by calculating a Cramer's phi-prime statistic, if this statistic was > 0.7, the variables were considered to be collinear.

Multivariate logistic regression with random effects was then performed; household was included as a random effect to account for the correlation of animals within households. As there were strong associations between many of the variables, all were initially included in the multivariate model to ensure any confounding relationships were identified, using a backwards step-wise procedure variables were permanently excluded from the final model when *p *> 0.05 the *p*-value for their association with *Brucella *serological status was greater than 0.05 and when removing the variable did not alter the odds ratios of the other variables by more than 20% or for variables which were protective, more than ±0.2. This analysis was then repeated using forward selection, starting with the variable with the lowest *p*-value from univariate analysis, to ensure the same results were obtained.

Data from the questionnaires and laboratory results were entered in Microsoft Office Excel 2007. Stochastic models were designed using @RISK 5.5 for Excel (Palisade Corporation Inc., Newfield, NY, USA) and statistical analysis of potential risk factors for *Brucella *spp. infection in cattle and buffalo was performed in STATA v.10 (STATA Corporation, Texas, USA).

## Results

### Village Studied

Households with cattle and buffalo in the village were highly homogenous with regards to husbandry and management-practices. Livestock were taken off the household during the day and secured on fields, generally used only by that household. Cattle and buffalo were milked twice a day and natural service was practiced throughout the village. Although cattle and buffalo from different households were kept separately there was the potential for contact between herds when they were being moved to and from the fields and when drinking from, and bathing in, water canals. Approximately a third (31.9%) of households kept sheep and/or goats which were mostly unsecured and could potentially contact animals from other household's herds.

### Households and Animals Studied

A total of 107 households and 155 individual animals were sampled; of these 109 (70.3%) were cows and 46 (29.7%) were buffaloes. Of the households initially selected, 29 (27.1%) did not have any cows or buffaloes and seven (6.54%) households had no lactating animals at the time of the visit, but there were no refusals to participate. Most households only had one lactating animal therefore the number of households sampled was exceeded in order to test more individual animals, however, target sample size for individual cattle and buffalo (166 animals) was not met.

Through sampling it was estimated that 78.7% of households kept cattle and/or buffaloes. Animals were generally kept in smallholdings; the median number of adult cows and buffalo in a household was three and the median number of calves and heifers in the household was two. Most (85.1%) animals sampled were born in the household and only 14 (13.1%) households had cows or buffaloes which were not born there, suggesting a low rate of transfer of animals between households.

### Seroprevalence of *Brucella *spp. in the Village

151 individual milk samples from 104 households were tested for antibodies against *Brucella *spp. (four samples were unsuitable). Twenty-two (14.6%) samples tested positive, of these 17 were from cows and five were from buffalo. Out of 104 households tested 20 (19.2%) had at least one animal that tested seropositive for *Brucella *spp. These values were used to model the individual seroprevalence of *Brucella *spp. in cattle and buffalo, the most likely seroprevalence estimate was 11.0% with a (95% CI: 3.06% to 18.4%). After combining the distributions for each household size in a weighted average, the most likely true household seroprevalence was estimated to be 15.5% (95% CI: 6.61% to 24.7%).

### Risk factors for *Brucella *spp. seropositive status

Three variables were retained in the multivariate model of risk factors for *Brucella *spp. (Table 2); number of calves and heifers in the household, species and the presence of sheep and/or goats in the household. There was some correlation between the explanatory variables; however none exhibited strong collinearity from the results of the phi-prime test.

**Table 2:** Risk factors associated with *Brucella *spp. serological status in large ruminants

Variable	OR (95% CI)	*p*-value
Presence of sheep/goats: (0 = no, 1 = yes)	6.32 (1.44-27.9)	0.02

Species: (0 = cow, 1 = buffalo)	1.91 (0.49-7.45)	0.35

Total calves and heifers:		
0	1	-
1	0.03 (0.002-0.04)	0.01
2	0.18 (0.04-0.42)	0.04
3	0.07 (0.02-0.23)	0.02
4+	0.21 (0.11-0.34)	0.03

In comparison to households with no calves or heifers, cattle and buffalo kept in a household with one, two, three and ≥four calves and/or heifers had 0.03 (95% CI: 0.002 to 0.04), 0.18 (95% CI: 0.04 to 0.42), 0.07 (95% CI: 0.02 to 0.2) and 0.21 (95% CI: 0.11 to 0.34) times the odds of testing seropositive against *Brucella *spp., respectively.

Cattle and buffalo kept in a household with sheep and/or goats had 6.32 (95% CI: 1.44 to 27.9) times the odds of testing seropositive for *Brucella *spp. compared to those kept in a household with neither, (p = 0.02). Removing species from the model altered the odds ratio for the association between testing seropositive for *Brucella *spp. and keeping sheep and goats in the household by more than 20%, therefore it was kept in the multivariate model. The value of rho, which is the proportion of the total variance in the model explained by the variance between households, was 0.07 (p = 0.42), indicating low correlation of individual animals within households.

### KAPs of brucellosis in the village

All households agreed to take part in the second part of the study assessing the KAPs of the rural residents, therefore a total of 214 participants (107 responsible for rearing livestock and 107 responsible for the processing of dairy products) were interviewed during the second visit. All participants responsible for rearing livestock were male, and all those responsible for processing dairy products were female.

### Knowledge of brucellosis

Of the 107 participants responsible for rearing animals 89 (83.2%) were sure they had heard of a "disease named brucellosis" and 18 (16.8%) believed they had heard of the disease but were not sure. All but one participant believed cows and buffalo could have the disease and 98.1%, 99.1%, 86.0%, 85.0% and 0.9% of participants were very confident that cattle, buffalo, sheep, goats and poultry can have brucellosis, respectively. With regards to the symptoms of disease in animals 5.6% of participants thought animals infected with brucellosis have no symptoms, most participants (94.4%) believed abortion and some (15%) thought drop in milk production were clinical signs. Four participants mentioned additional symptoms such as fever, loss of appetite and loss of body weight.

**Figure 1:**
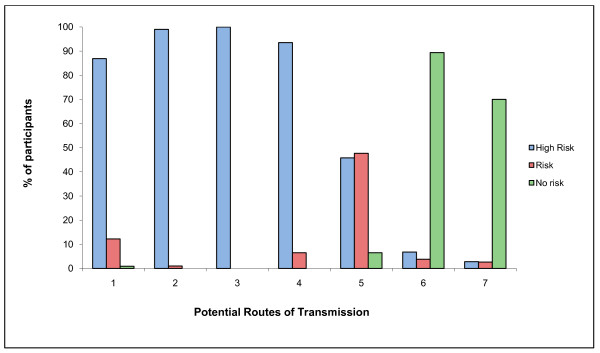
**Participant's opinions regarding routes of infection of humans with *Brucella *spp**. Where 1 is physical contact with infected animals, 2 is assisting in the parturition of infected animals, 3 is contact with foetuses or foetal membranes of infected animals, 4 is drinking raw milk from infected animals, 5 is consuming cheese made from milk of infected animals, 6 is consuming meat from infected animals and 7 is contact with infected people.

103 (96.3%) participants correctly believed brucellosis is transmissible from animals to humans. Of those interviewed 95 (88.8%) thought *Brucella *spp. can be transmitted to humans through physical contact with animals, 100% believed it can be transmitted through contact with foetuses or foetal membranes, 99 (92.5%) believed it can be transmitted through drinking contaminated milk and no participants believed it could be transmitted via contact with infected humans (Figure 1).

### Farmer's attitudes and practices with regards to brucellosis

101 (94.4%) participants stated that most people in the village assist with calving, usually by pulling the calf out or removing foetal membranes and 102 (95.3%) participants thought most people in the village assist in the parturition of sheep and goats. All participants agreed that most farmers dispose of placentas and aborted foetuses in the water canals, and the dumping of animal carcases in water canals was observed whilst sampling the village. In addition all participants believed that villagers never wear protective gloves or masks when assisting with the parturition or abortion of animals or whilst handling placentas and aborted foetuses, which was also observed during the fieldwork.

**Figure 2:**
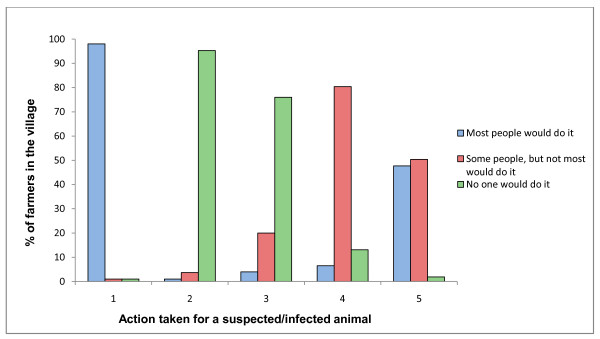
**Participant's opinions regarding management of *Brucella *suspected/infected large ruminants by most farmers**. Where 1 is ask the local veterinarian for advice, 2 is buy a vaccine or treatment, 3 is sell the animal to their neighbour, 4 is sell the animal at market and 5 is sell the animal to the butcher.

Participants' opinions regarding the action most farmers take when they have an animal infected, or suspected to be infected, with *Brucella *spp. are presented in Figure 2. Eighty-one participants (78%) agreed that no one would sell a suspected or infected animal directly to their neighbours, but 86 (80.4%) believed some farmers would sell the animal at market. Most participants agreed that either some (47.7%), or most (50.5%), farmers would sell a suspected or infected animal directly to the butcher; in addition almost all (98%) thought most farmers would call the local veterinarian.

**Figure 3:**
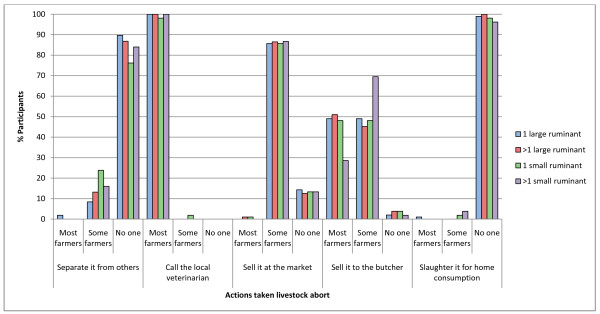
**Results from the interviews with livestock owners regarding management of different species which have aborted**. Results from the interviews with livestock owners in the village regarding the management of abortions in different ruminant species.

Figure 3 shows the management of animals that abort according to participants' opinions. 95 (89.7%) participants believed farmers would never separate a cow or buffalo and 80 (76.2%) participants thought farmers would never separate a sheep or goat that has aborted from the rest of their herd. If farmers had more than one aborting sheep or goat even more participants (84.0%) believed most farmers would not separate them from their other animals. Almost all participants believed most farmers (49%) or some farmers (49%) would sell a cow or buffalo which has aborted to the butcher. Most participants (85.7%) believed some farmers would sell cattle, buffalo, sheep and goats which have aborted at markets, even if more than one animal aborted, however all participants agreed that most farmers in the village would call the local veterinarian for advice.

### Processing and consumption of dairy products

Approximately a third (32.7%) of households regularly sold their raw milk, however, most participants processed their animal's milk into cheese (82.2%), cream (86.9%) and/or butter (87.9%). All participants said they boiled raw milk before consumption but no participants' boiled milk before processing it into other dairy products.

## Discussion

The current study adopted an integrative approach to brucellosis research; investigating the disease from both a veterinary and human health stand point. Individual and household seroprevalence of *Brucella *spp. in cattle and buffalo was estimated to be 11.0% (95% CI: 3.06% to 18.4%) and 15.5% (95% CI: 6.61% to 24.7%), respectively, confirming that brucellosis was endemic in the studied village. Although, caution should be taken when interpreting point estimates due to their low precision, reflected in the wide confidence intervals. The study also identified a potential role for sheep and goats in the transmission of *Brucella *spp, to large ruminants and went on to look at how human behaviour may influence the spread of the disease, both between animals and from animals to humans.

Unbiased estimates of seroprevalence of brucellosis are lacking in Egypt and therefore comparisons with estimates of *Brucella *spp. seroprevalence from other studies have to be made with caution. However, they are higher than those obtained in previous studies in different governorates of Egypt by Samaha et al in 2008, who estimated an overall seroprevalence of 5.44% in cattle and 4.11% in buffaloes [13].

Although animals were tested for antibodies against *Brucella *spp. as oppose to *Brucella *spp. organisms, recovery from *Brucella *infection in ruminants is rare and animals usually remain infected for life therefore it is likely that seropositive animals were still infected [24]. Therefore, the seroprevalence estimates provide information on the proportion of cows and buffalo in the village which are potentially shedding *Brucella *organisms in milk and during parturition, posing a public health threat. If any cows or buffaloes in the study were vaccinated against *Brucella *then seroprevalence may have been overestimated but the veterinarian, who had worked in the village for many years, was certain that no animals in the village were vaccinated.

One limitation to the current study is that the organism large ruminants were infected with was not isolated and typed, however, *B. melitensis *is the predominant *Brucella *species in Egypt [10,13]. In addition keeping sheep and goats in the household was the primary risk factor for cattle and buffalo testing serologically positive for *Brucella *spp., which is consistent with other studies in areas where *B. melitensis *is the predominant species present, therefore it is assumed that seropositve cattle and buffalo were infected with this species [13,25]. Species (cattle vs. buffalo) was acting as a negative confounder for the association between *Brucella *spp. and keeping sheep or goats in the household; buffaloes appear to be less likely to be seropositive than cows, however, households which had buffaloes tended to be larger and hence were more likely to have sheep and goats. The association between species and *Brucella *spp. was probably not significant due to the low numbers of buffaloes sampled in the study.

Highly homogeneous production management systems were observed in the village, which may explain why no other risk factors for an animal being seropositive for *Brucella *spp. were identified. Alternatively, perhaps there is little transmission of the disease between cattle and buffalo and maintenance of *Brucella *spp. in these species is entirely dependent on the small ruminant population in the village. This study focussed on brucellosis in large ruminants and no sheep or goats were tested in the village. Although it would have been useful to test sheep and goats for *Brucella *spp., a recent study, in 40 villages from nearby Kafrelsheikh governorate estimated that the village flock of small ruminants was infected in more than 60% of villages, village flocks are mobile and used for breeding with household animals. In the same study overall true prevalence of *Brucella *spp. in sheep and goats, was estimated to be 13.5% (95% CI: 9.3% to 17.7%) and 12.5% (95% CI: 8.6% to 16.4%), respectively [12]. These estimates indicate that *Brucella *spp. is likely to be present in small ruminants in the village, possibly at a high level.

The high level of awareness of the disease in the village is consistent with an endemic situation. Most participants responsible for rearing livestock were aware of brucellosis and were knowledgeable about the susceptibility of different animal species. There appeared to be a higher awareness of the disease in cattle and buffalo than sheep and goats, which may be because more households kept cattle or buffalo (~80%) compared to sheep or goats (~30%). Also, as an abortion in a cow or buffalo is likely to have a greater economic impact on the household than an abortion in sheep or goats, there may be more awareness of disease events in large ruminants. Participants had very accurate knowledge of the main clinical signs of brucellosis in ruminants and the transmission pathways from animals to humans.

Despite the high degree of awareness and accurate knowledge of the disease, its transmission and its effects, most people would not separate animals that aborted from other household animals. This is one of the major risk factors for disease transmission between animals as susceptible animals can be infected via contact with infected animals or contact with aborted materials or products of parturition [8]. This probably demonstrates the lack of facilities for isolation of suspected and/or infected animals in the current smallholder system. Furthermore, in such a hyperendemic setting and given that infection is often subclinical and cattle and buffalo may not always abort, livestock owners may accept that the disease is widespread and that infection is rarely avoided by separating their aborting or calving animals from the rest of their herd.

According to the results farmers may sell animals which abort to the butcher, if ruminants infected with *Brucella *spp. are often sent for slaughter this may mean abattoir workers may be at a high risk of occupational infection with *Brucella *spp. The results also indicate that some farmers may sell animals in markets if they believe they are infected with *Brucella *spp. This may increase the transmission of brucellosis, not only between households in the same village, but also between villages and even larger geographical areas as animals purchased at a market can be moved without restriction to anywhere in Egypt.

When these issues were discussed with participants during the interview most mentioned that it is easier to sell animals on than to notify the veterinary authorities and wait until they test and slaughter the positive animal. This is also more economical as, according to the participants and veterinarians, the compensation received is less than 20% of the market value of the animal and often takes more than a year to receive. A previous study by Pappas et al (2006) investigating patient perceptions of brucellosis in Greece found that around 44% of patients with brucellosis would not allow veterinary investigation as they were worried about the effects on their herd. This indicates that underreporting is likely to be a problem hindering brucellosis control in other areas.

Although farmers are unlikely to report suspected cases to the authorities, they will usually contact the local veterinarian, therefore it seems local veterinarians may not be reporting brucellosis. In the authors experience (MME and YMH), veterinarians will advise households to fatten suspected animals in order to send them to slaughter and will not advise the farmers to get the animal tested, however, they will leave the final decision to the farmer. Veterinarians usually reside in within the village they work in and there appears to be a strong sense of loyalty between them and livestock owners; if they know the farmer, who is often a friend or even a relative, will not get full market value for their animal in compensation there is no incentive for them to report the farmer, especially as they are unlikely to receive any penalty for not reporting. Most local veterinarians are affiliated with the government and there is the opportunity for the government to work more closely with these veterinarians in order to improve the flow of information between themselves and livestock owners. It is likely the only way livestock owners and veterinarians will begin reporting the disease is if adequate compensation or replacement animals are received.

Cattle and buffalo infected with *Brucella *spp. excrete high concentrations of the organism in their milk, placental membranes and aborted foetuses [8,9]. Therefore there is a risk of humans becoming infected via direct contact with their animals and through consumption of their dairy products. Most people in the village assist in the parturition and abortion of ruminants (cattle, buffalo, sheep and goats) and handle foetal membranes and aborted foetus without wearing any protective gloves or masks, even though most are aware these are high-risk activities. Placentas and aborted foetuses are disposed by most people into water canals, which can be a source of infection since most animals in this area had access to the water canals for drinking and bathing. *B. melitensis *has recently been isolated from catfish in water canals in the area indicating that the water is heavily polluted by animal waste [26]. This may also present a new potential route of human infection. In addition, other species may be involved in the transmission of the disease in the village; *Brucella melitensis biovar 3 *(which is the most common isolate of *Brucella *spp. in Egypt) has previously been isolated from dogs and rats [27]. Seroprevalence in these species can be higher close to positive herds [27]; in Damitta governorate all three dogs and 14.3% (10 out of 70) rats trapped near a large seropositive dairy herd were also *Brucella *spp. seropositive. Rats are often found near canals and dogs also use these canals for bathing, therefore these species could aid the spread of *Brucella *spp [28].

With regard to the risk of human exposure to *Brucella *spp. via drinking milk, the results suggest this is negligible since all participants' boiled raw milk before consumption. However, there seems to be a potential risk of exposure from other dairy products processed and consumed regularly in more than 80% of households, such as cheese homemade from raw milk. In the Egyptian governorate of Damietta, *Brucella melitensis *biovar 3 has been isolated from soft white cheese and yoghurt on sale in dairy shops, the products were made from cow's milk which was not heat treated [29].

Although the results obtained for this village cannot be generalised to other villages in the area, there was no prior information regarding brucellosis that could have directed the study to this village because of a high seroprevalence. Brucellosis is considered endemic throughout Egypt and husbandry practices are similar to those observed in this village, it is therefore possible that the epidemiology of *Brucella *spp. may be similar in other rural areas [3,10].

The difficulty of implementing a comprehensive brucellosis control programme in Egypt has been highlighted in previous reports [10,11]. These results add to the body of evidence that have identified a critical role of small ruminants in the maintenance and transmission of brucellosis in large ruminants and realistic interventions targeted at small ruminant brucellosis are needed. Until decisive effective action is taken to reduce the incidence of ruminant brucellosis, the local population will have a high risk of exposure.

Public health education could contribute to risk mitigation and should focus on cost-effective strategies to reduce occupational exposure and consumption of unpasteurized dairy products. All participants were interested in the public health education the interviewer provided and some suggested this should be presented as a poster in public places, the interviewer was also asked to give public talks in order to disseminate information to the whole community. This is a good indication that there is potential for promotion of improved husbandry and dairy processing practices that could reduce the risk of exposure, not only to *Brucella *spp, but also to other zoonotic pathogens [30]. Recommendations should be achievable and take into account that the livelihoods of most rural residents depend on the few animals they own.

## Conclusions

Brucellosis is endemic at high levels among the large ruminant population of the studied village in the Nile Delta region of Egypt, where the main risk factor for cattle and buffalo seropositive status is the presence of sheep or goats in the same household. Animals suspected of having brucellosis are much more likely to be sold to the butcher or in the market than reported to the necessary authorities. Given the observed seroprevalence and reported practices, in the studied population, there is a high risk of occupational exposure of *Brucella *spp. in livestock keepers, whilst assisting in parturition or abortion of ruminants. There is also the potential for human exposure through consumption of dairy products processed from raw milk that are regularly consumed in most households.

## Competing interests

The authors declare that they have no competing interests.

## Authors' contributions

HH designed the first questionnaire, carried out the fieldwork for the first household visits, analysed the first part of the study and drafted the manuscript. ME designed the second questionnaire and translated it into Arabic, analysed the second part of the study and participated in the write-up of the manuscript. YH and JG contributed to the concept, design and analysis of the study and JG critically revised the manuscript. WE and AT supervised the laboratory work in Egypt and helped with the fieldwork. All authors read and approved the final manuscript.

## Pre-publication history

The pre-publication history for this paper can be accessed here:

http://www.biomedcentral.com/1471-2458/11/341/prepub
